# *In Vitro* TNF-**α** Inhibitory Activity of Brazilian Plants and Anti-Inflammatory Effect of* Stryphnodendron adstringens* in an Acute Arthritis Model

**DOI:** 10.1155/2016/9872598

**Published:** 2016-10-27

**Authors:** Bárbara O. Henriques, Olívia Corrêa, Elaine Patrícia C. Azevedo, Rodrigo M. Pádua, Vívian Louise S. de Oliveira, Thiago Henrique C. Oliveira, Daiane Boff, Ana Carolina F. Dias, Danielle G. de Souza, Flávio A. Amaral, Mauro M. Teixeira, Rachel O. Castilho, Fernão C. Braga

**Affiliations:** ^1^Department of Pharmaceutical Sciences, Faculty of Pharmacy, Universidade Federal de Minas Gerais, Campus Pampulha, Avenida Antônio Carlos 6627, 31.270-901 Belo Horizonte, MG, Brazil; ^2^Department of Biochemistry and Immunology, Institute of Biological Sciences, Universidade Federal de Minas Gerais, Campus Pampulha, Avenida Antônio Carlos 6627, 31.270-901 Belo Horizonte, MG, Brazil; ^3^Department of Microbiology, Institute of Biological Sciences, Universidade Federal de Minas Gerais, Campus Pampulha, Avenida Antônio Carlos 6627, 31.270-901 Belo Horizonte, MG, Brazil

## Abstract

*Stryphnodendron *species, popularly named “barbatimão,” are traditionally used in Brazil as anti-inflammatory agents. This study aimed to investigate the effect of barbatimão and 11 other species on the production of tumor necrosis factor-alpha (TNF-*α*) in lipopolysaccharide- (LPS-) stimulated THP-1 cells, as well as their anti-arthritis activity. The extracts of* Stryphnodendron adstringens*,* Stryphnodendron obovatum*,* Campomanesia lineatifolia*, and* Terminalia glabrescens* promoted a concentration-dependent inhibition of TNF-*α*. Mice injected with LPS in the knee joint were treated* per os* with fractions from the selected extracts. Both the organic (SAO) and the aqueous (SAA) fractions of* S. adstringens *promoted a dose-dependent reduction of leukocyte migration and neutrophil accumulation into the joint, but none of them reduced CXCL1 concentration in the periarticular tissue. In contrast, treatment with* C. lineatifolia *and* T. glabrescens* fractions did not ameliorate the inflammatory parameters. Analyses of SAO by Ultra Performance Liquid Chromatography (UPLC) coupled to electrospray ionization mass spectrometry (ESI-MS) led to the identification of gallic acid along with 11 prodelphinidins, characterized as monomers and dimers of the B-type. Our findings contribute to some extent to corroborating the traditional use of* S. adstringens* as an anti-inflammatory agent. This activity is probably related to a decrease of leukocyte migration into the inflammatory site. Polyphenols like gallic acid and prodelphinidins, identified in the active fraction, may contribute to the observed activity.

## 1. Introduction 

Tumor necrosis factor-alpha (TNF-*α*) is a key proinflammatory cytokine rapidly released after inflammatory stimuli. It prompts several intracellular events that results in the activation of the transcription nuclear factor kappa B (NF-*κ*B), leading to the production of other proinflammatory cytokines, chemokines, and proteases [1, 2]. The overproduction of this cytokine is associated with different inflammatory diseases, including rheumatoid arthritis [3]. TNF-*α* inhibitors are clinically useful for treating rheumatoid arthritis; however, the available inhibitors are costly biological drugs that demand parenteral administration and are not effective to all patients [4]. Therefore, TNF-*α* is considered a valid target for the development of new drugs to treat chronic inflammatory diseases and the identification of compounds that may act as antagonists of this cytokine by oral route is currently demanded.

The anti-inflammatory properties of natural products of different chemical classes have been demonstrated by* in vitro* and* in vivo* experiments. Plant constituents like flavonoids, terpenoids, alkaloids, cannabinoids, ginsenosides, and phytosterols have been reported to inhibit the upstream signaling molecules that are involved in TNF-*α* expression, as reviewed by Verma et al. [5] and Iqbal et al. [6]. Moreover, some natural products with TNF-*α* inhibiting property also exhibit antioxidative stress activity [7].

The Brazilian flora is a vast source of bioactive molecules and we have recently reported the screening of several medicinal plants in the search for new TNF-*α* inhibitors [8, 9]. Further investigation of some selected species resulted in the isolation and/or characterization of different TNF-*α* antagonists, including two new glucosylated heterotrimeric flavonoids from* Mansoa hirsuta* [10], along with flavone C-glycosides and aconitic acids from* Echinodorus grandiflorus* [11]. In our ongoing search for new TNF-*α* inhibitors, in the present study we report the inhibition of cytokine release by lipopolysaccharide- (LPS-) stimulated THP-1 monocytic cells elicited by the extracts of 13 Brazilian medicinal plants, along with the activity of* Stryphnodendron adstringens* on an acute arthritis model, as well as its chemical characterization.

## 2. Materials and Methods 

### 2.1. Plant Materials and Extracts Preparation

Thirteen plant species were selected for study based on their traditional uses to treat inflammatory diseases. The collection and extraction data along with the ethnopharmacological uses of the selected plants are described in Table 1.

The plant materials were dried at 40°C in a ventilated oven and powdered in a knife mill. Portions (5 g) of the powdered plant materials were extracted with ethanol 96°GL at room temperature, in an ultrasound bath (3 × 50 mL, 20 min). The extracts were filtered and the solvent removed by evaporation under reduced pressure on a rotatory evaporator, at maximal temperature of 50°C, resulting in the crude extracts depicted in Table 2.

A similar procedure was adopted to prepare the dichloromethane and the ethyl acetate extracts of the most active plants (*Campomanesia lineatifolia*,* Stryphnodendron adstringens*,* Stryphnodendron obovatum*, and* Terminalia glabrescens*). In contrast, the aqueous extracts were prepared by infusion (leaves) or decoction (bark), employing 5 g of the vegetal drug to 50 mL of distilled water. The obtained extracts were filtered and dried by lyophilization. The yields (weight/weight w/w) of the dichloromethane, ethyl acetate, and aqueous extracts were, respectively, 7.70%, 10.04%, and 25.40% to* C. lineatifolia, *6.18%, 8.58%, and 38.00% to* S. adstringens, *6.67%, 9.54%, and 35.80% to* S. obovatum*, and 11.84%, 13.17%, and 19.56% to* T. glabrescens.*

### 2.2. TNF-*α* Inhibition Assay

The inhibition of TNF-*α* release elicited by the extracts (Table 1) was assayed on LPS-stimulated THP-1 cells (ATCC TIB-202), as previously described [27]. In summary, the cells (1.0 × 10^6^ cells/mL) were transferred to a 96-well microplate at a density of 100,000 cells per well and incubated for 18 h. Then, they were pretreated with the extracts and fractions for 3 h and LPS (Sigma-Aldrich, USA) was added as inflammatory stimulus (100 ng/mL, 20 *μ*L). After incubation at 37°C overnight, the plate was centrifuged (1800 ×g, 5 min, 16°C), the supernatant was collected, and TNF-*α* was quantified by the cytokine-specific sandwich quantitative enzyme-linked immune-sorbent assay (ELISA), according to the manufacturer's instructions (TNF-alpha duo set, DY210, R&D Systems, USA). Cell pellet was used to evaluate cell viability by the 3-(4,5-dimethylthiazol-2-yl)-2,5-diphenyltetrazole (MTT) method. Cell viability was evaluated for all tested samples (extracts and fractions) and was determined from the ratio between viable cells and total cells, using untreated cells as reference for viability. Samples that gave cell viability above 90% were considered nontoxic for the THP-1 cell line.

The inhibition of TNF-*α* release by LPS-stimulated THP-1 cells was calculated by the ratio between the TNF-*α* amount secreted by treated cells (pg/mL) and the level of this cytokine (pg/mL) observed for nontreated cells stimulated with LPS. THF-*α* inhibition was reported as percentage values [1 − (cytokine secretion of treated cells/cytokine secretion of cells cultivated with solvent control) × 100]. The statistical significance of differences was calculated employing GraphPad Prism software, version 5.0 (GraphPad Software Inc., USA), using one-way ANOVA followed by Newman-Keuls posttest for multiple comparisons. Results were considered significantly different when *p* < 0.05.

The samples were tested in triplicate at the concentrations of 62.5, 125, and 250 *μ*g/mL. For* C. lineatifolia, S. adstringens, S. obovatum*, and* T. glabrescens*, the extracts prepared with dichloromethane, ethyl acetate, and water were also tested at the same concentrations. Dexamethasone (Sigma-Aldrich, 0.1 *μ*M) was employed as positive control.

### 2.3. Preparation of Organic and Aqueous Fractions from the Crude Extracts

The ethanolic extracts of* C. lineatifolia, S. adstringens*, and* T. glabrescens* were fractionated by partition between immiscible solvents. Portions (3.0 g) of each extract were suspended in water (54 mL) and partitioned with a mixture of ethanol/*i*-propanol/*n*-butanol (42 : 12 : 6 volume/volume/volume, v/v/v; 180 mL), under mechanical stirring for 30 min. The phases were separated in a separation funnel, and the aqueous layer was collected and subjected to extraction with the organic mixture twice. The organic and aqueous phases were concentrated to residue under reduced pressure in a rotatory evaporator at maximal temperature of 50°C, resulting in the organic and aqueous fractions of* C. lineatifolia* (1.50 g and 1.19 g, resp.),* S. adstringens *(1.32 g and 1.27 g, resp.), and* T. glabrescens* (1.74 g and 0.96 g, resp.).

### 2.4. LPS-Induced Acute Inflammation 

#### 2.4.1. Animals and Experimental Protocol

The organic and aqueous fractions of* C. lineatifolia, S. adstringens*, and* T. glabrescens* had their anti-inflammatory activity assayed, as described by Cunha et al. [28]. Female Swiss mice (6 weeks old), weighing between 28 and 32 g, obtained from the Animal Housing Unit of the Faculty of Pharmacy, UFMG, were employed in the experiments. All experiments received prior approval from the UFMG Ethics Committee (CETEA, certificate number 83/2015). The animals (6 per group) were treated with the fractions (10, 100, and 1000 mg/kg,* per os*) or the positive control (dexamethasone, 4 mg/kg, subcutaneous) or the vehicle (water containing 0.5% weight/volume, w/v, carboxymethyl cellulose). Ninety min after treatment, they were anesthetized by intraperitoneal injection of a mixture of ketamine and xylazine in phosphate-buffered saline (PBS) (1 : 0.5 : 3 v/v/v) and 10 *μ*L of a LPS solution (10 *μ*g/mL in saline) was injected into the knee joint (100 ng/articular cavity). Control animals were injected with 10 *μ*L of PBS. After 12 h, the articular cavity was exposed and washed twice with 5 *μ*L of 3% bovine serum albumin (BSA) in PBS. The joint lavage was diluted to 100 *μ*L using 3% BSA and employed for total and differential cell counting.

The total cell count was performed using an aliquot (10 *μ*L) of the joint lavage stained with Turk solution (10 *μ*L), followed by counting in a Neubauer chamber. For the neutrophil count, slides were prepared by centrifuging the remaining 90 *μ*L of the joint lavage at 450 ×g for 5 min in a cytocentrifuge (Shandon Cytospin 3, Thermo Scientific, USA). The air-dried smears were stained with panoptic stain. The periarticular tissue was removed for the determination of chemokine (C-X-C motif) ligand 1 (CXCL1) and TNF-*α* levels, along with myeloperoxidase (MPO) activity.

#### 2.4.2. Determination of CXCL1 and TNF-*α* by Immunoassay and MPO Activity

The periarticular tissue was weighed and extracted with a cytokine extraction solution (0.1 mM phenylmethylsulfonyl fluoride (PMSF), 0.1 nM benzethonium chloride, 10 mM ethylenediaminetetraacetic acid (EDTA), 20 KI aprotinin A, and 0.05% Tween 20 in PBS) at the ratio of 1 mL of solution to 100 mg of tissue. The material was homogenized and centrifuged at 10.000 ×g for 10 min, at 4°C. The supernatant was collected and TNF-*α* and CXCL1 levels were measured using ELISA according to the manufacturer's instructions (CXCL1 duo set, DY453, and TNF-alpha duo set, DY410, R&D Systems, USA).

The cell pellet was reserved for the determination of myeloperoxidase activity as previously described [29]. It was suspended in Buffer 2 (0.05 M NaPO_4_ and 0.5% w/v* n*-hexyltrimethylammonium bromide, pH 5.4) and homogenized for 30 s. The samples were frozen and unfrozen three times in liquid nitrogen, centrifuged at 10,000 ×g for 15 min, at 4°C, and the supernatant was collected for the determination of MPO activity. An aliquot (25 *μ*L) of the samples, diluted three times in Buffer 2, was added to a 96-well microplate. Then, 25 *μ*L of 1.6 mM 3,3′,5,5′-tetramethylbenzidine (TMB) solution was added and the plate was incubated at 37°C for 5 min. After that, 0.002% hydrogen peroxide (100 *μ*L) was added and the plate was incubated at 37°C for additional 5 min. The reaction was stopped by adding 50 *μ*L of 1.0 M sulphuric acid solution. MPO activity was determined by measuring the absorbance at 450 nm in a microplate reader (Infinite 200 Pro, Tecan, Switzerland). The MPO activity was calculated by comparison of the optical density (OD) of the samples with the OD obtained for neutrophils from mice lungs submitted to the same procedure and the results were expressed as MPO relative units/*μ*g.

#### 2.4.3. Determination of TNF-*α* by Real-Time Polymerase Chain Reaction (RT-PCR)

Expression of TNF-*α* transcripts in periarticular tissue was also determined by real-time PCR, as previously described [30]. Total RNA was isolated from the synovial tissue using Trizol (Ambion, Life Technologies, USA) following manufacturer's instructions. Total RNA was resuspended in diethylpyrocarbonate-treated water and stocked at −70°C. Preparation of cDNA was performed using 1 *µ*g of RNA and the SuperScript™ III Reverse Transcriptase kit (Invitrogen, Life Technologies, USA), according to manufacturer's instructions. Real-time PCR was performed using the Power SYBR Green PCR Master Mix 2x (Applied Biosystems, USA) on a 7500 Fast Real-Time PCR System (Applied Biosystems, USA). Relative expression of the analyzed genes was determined by ΔΔCt method, whereby data for each sample were normalized to glyceraldehyde 3-phosphate dehydrogenase (GAPDH) constitutive gene and expressed as a fold change compared with control [30]. The following primer pairs were used: for GAPDH, 5′- ACG GCC GCA TCT TCT TGT GCA -3′ (forward) and 5′- CGG CCA AAT CCG TTC ACA CCG A -3′ (reverse); for mTNF-*α*, 5′- ACG GCA TGG ATC TCA AAG AC -3′ (forward) and 5′- AGA TAG CAA ATC GGC TGA CG -3′ (reverse).

#### 2.4.4. Statistical Analysis

The results were expressed as mean ± SEM of six animals for each group. The statistical significance of differences was calculated employing the software GraphPad Prism, version 5.0 (GraphPad Software Inc., USA), using one-way ANOVA followed by Newman-Keuls posttest for multiple comparisons. Results were considered significantly different when *p* < 0.05.

### 2.5. Characterization of* S. adstringens* Chemical Composition by Ultra Performance Liquid Chromatography (UPLC) Coupled to Electrospray Ionization Mass Spectrometry (ESI-MS)

The organic fraction of* S. adstringens *stem bark (SAO) was analyzed by UPLC-ESI-MS. The analyses were carried out on an ACQUITY Ultra Performance LC system (Waters, USA) linked to a PDA 2996 photo diode array detector (Waters, USA) and an ACQUITY TQ detector (Waters MS Technologies, UK), equipped with a Z-spray electrospray ionization (ESI) source operating in negative and positive mode. MassLynx software (version 4.1, Waters, USA) was used to control the instruments, as well as for data acquisition and processing.

The analyses were carried out on a LiChrospher 100 RP-18 column (250 × 4 mm i.d., 5 *μ*m; Waters) at 40°C, eluting with a linear gradient of water (A) and acetonitrile (B), both containing 0.1% v/v formic acid, at a flow rate of 1 mL/min, as follows: 5% to 40% of B in 60 min, followed by 5 minutes of isocratic elution (40% of B); and return to the initial conditions in 5 minutes [31]. A reequilibration time of 5 min was kept between runs. The sample solutions (5 mg/mL for the fraction and 1 mg/mL for the reference compounds) were dissolved in methanol/water (1 : 9 v/v), filtered over 0.45 *μ*m polytetrafluoroethylene (PTFE) membrane, and aliquots (8 *µ*L) were automatically injected into the system. The chromatograms were registered at *λ* 210 nm.

The MS operating parameters were as follows: scan mode negative and positive within the mass range of* m/z* 100–2000 Da, desolvation temperature of 375°C, desolvation gas flow of 600 L/h, cone gas flow of 60 mL/min, source temperature of 120°C, cone voltage of 60 V, and capillary voltage of 3000 V. The nebulizer gas was nitrogen.

Tandem mass spectrometric (UPLC-ESI-MS/MS) analyses were carried out on a BEH RP-18 column (50 × 2.1 mm i.d., 1.7 *μ*m; Waters) at 40°C. A linear gradient of water (A) and acetonitrile (B), both containing 0.1% v/v formic acid, was employed, at a flow rate of 0.3 mL/min, 5 to 95% of B in 10 min, followed by 1 min of isocratic elution, returning back to the initial conditions in 2 min. A two-minute reequilibration time was kept between runs. The following MS/MS operating parameters were used for the analyses: scan mode negative and positive within the mass range of* m/z* 100–1500, desolvation temperature of 350°C, desolvation gas flow of 550 L/h, cone gas flow of 50 mL/min, source temperature of 120°C, cone voltage of 60 V, and capillary voltage of 3500 V. Nitrogen and argon were employed as nebulizer gas.

## 3. Results and Discussion 

### 3.1. Inhibition of TNF-*α* Release by THP-1 Cells Elicited by the Extracts

The plant extracts were evaluated for their toxicity on THP-1 cells by the MTT method to assure that the anti-TNF-*α* activity was not resulting from toxic effects. All extracts were considered noncytotoxic, with the exception of* Mikania glomerata *(stems) and* Cordia guazumaefolia *(leaves and stems), which produced cell viability below 90% at the highest tested concentration (250 *μ*g/mL).

The effect of the noncytotoxic extracts on TNF-*α* release by LPS-stimulated THP-1 cells was assayed at three concentrations and the results are depicted in Table 2. The extracts of* Licania tomentosa, Paepalanthus bromelioides*,* Vitex polygama*,* Bowdichia virgilioides *(leaves and stems), and* Campomanesia lineatifolia *(stems) enhanced the production of TNF-*α* by the LPS-stimulated cells, suggesting the presence of proinflammatory constituents. Interestingly, when assayed in nonstimulated THP-1 cells, none of the extracts increased TNF-*α* production (data not shown). These plant extracts probably act synergistically with LPS, increasing the inflammation response.

The seeds and barks of* B. virgilioides* are traditionally used to treat different inflammatory conditions [12]. Although* B. virgilioides* enhanced the production of THF-*α* in the screening here reported, the anti-inflammatory properties of extracts prepared with stem barks and leaves of the species have been previously demonstrated in animal models, using carrageenan and acetic acid as inflammatory stimuli [32, 33]. In the same direction, a decoction from the leaves of* L. tomentosa *was shown to have antinociceptive and anti-inflammatory properties [34].

**Figure 1:**
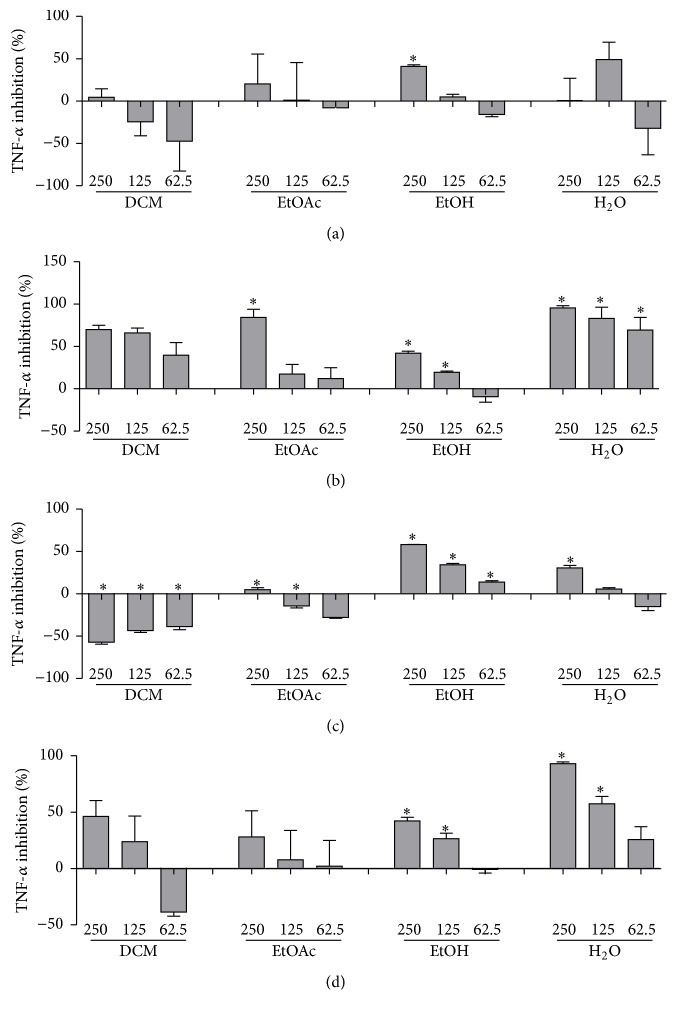
Inhibition of TNF-*α* release by LPS-stimulated THP-1 cells elicited by plant extracts, assayed at 62.5, 125, and 250 *µ*g/mL: (a)* Campomanesia lineatifolia *(leaves), (b)* Stryphnodendron adstringens*, (c)* Stryphnodendron obovatum,* and (d)* Terminalia glabrescens *(leaves). Data represent the mean inhibition (%) ± standard deviation (SD) from three separate experiments. Means were analyzed by one-way ANOVA, following multiple comparison by Newman-Keuls test (*p* < 0.05), compared with control (cells + LPS). *∗* indicates significant inhibition of TNF-*α* release in comparison to LPS-stimulated cells; *p* < 0.05.

Among the 19 extracts assayed, those of* C. lineatifolia* (leaves),* Terminalia glabrescens* (leaves),* Stryphnodendron adstringens *(bark),* Stryphnodendron obovatum* (bark),* Hymenaea stigonocarpa*, and* Vernonia phosphorea* inhibited the release of TNF-*α* in a concentration-dependent manner. The extract of* Hymenaea courbaril *also inhibited the cytokine release, but the response could not be characterized as concentration-dependent, since there was no difference in the inhibition elicited by the lower and the higher concentrations assayed. Aiming at further investigating the anti-TNF-*α* effect of some of the active species, extracts of increasing polarities (dichloromethane, ethyl acetate, and water) were prepared from* C. lineatifolia* (leaves),* T. glabrescens* (leaves),* S. adstringens *(bark), and* S. obovatum* (bark) and their effect on cytokine inhibition was evaluated on LPS-stimulated THP-1 cells (Figure 1).

The ethanolic and aqueous extracts of* S. adstringens*,* S. obovatum*, and* T. glabrescens *inhibited TNF-*α* production significantly at most of the assayed concentrations, whereas the ethyl acetate extract of* S. adstringens *was active solely at 250 *µ*g/mL. In contrast, the extracts of* S. obovatum *prepared with dichloromethane and ethyl acetate seem to present proinflammatory properties, since a significant increase in TNF-*α* concentration was observed after treatment. Therefore, only the polar extracts of these species elicited significant inhibition of TNF-*α* production by stimulated cells.

Similarly to our results, Nishijima [35] reported significant TNF-*α* inhibition promoted by high polar extracts from* Rhamnidium elaeocarpum* stem bark on murine macrophages stimulated by LPS. Likewise, the TNF-*α* inhibitory activity of ethanolic extracts from the Brazilian plants* Caryocar brasiliense, Casearia sylvestris*, and* Coccoloba cereifera *was recently described by us [8]. On the other hand, other authors reported higher TNF-*α* inhibitory activity for low polar extracts in comparison to methanol extracts, like those prepared with dichloromethane and chloroform from Bhutanese plants [36].

This is the first report on the anti-TNF-*α* activity of* C. lineatifolia*, although other species of* Campomanesia* have been described to have anti-inflammatory properties, like* Campomanesia velutina*, whose ethanolic extract from leaves decreased the paw edema induced by carrageenan in mice [37]. In a previous publication, we described the anti-TNF-*α* activity of* Terminalia glabrescens* on LPS-stimulated THP-1 cells [8]. The inhibitory activity here reported for an extract prepared with the plant material collected at a distinct site confirmed our previous finding and disclosed* T. glabrescens* as a promising species for* in vivo* studies, reported in Section 3.2.

### 3.2. Evaluation of Selected Fractions on LPS-Induced Acute Arthritis

The initial screening of the extracts on LPS-stimulated THP-1 cells disclosed* S. adstringens*,* S. obovatum*,* C. lineatifolia*, and* T. glabrescens* with significant TNF-*α* inhibiting activity. The aqueous extracts of* S. adstringens *and* T. glabrescens* elicited more potent anti-TNF-*α* responses on THP-1 cells than their ethanolic extracts; however, in view of the higher complexity of the water extracts and the difficulties for their chemical characterization, they were not prioritized for further studies. Hence, the ethanolic extracts of the above-mentioned species, except* S. obovatum*, were fractionated by partition between immiscible solvents and the resulting organic and aqueous fractions were evaluated in the LPS-induced acute arthritis model, at three different doses (10, 100, and 1000 mg/kg).

The organic and the aqueous fractions of* C. lineatifolia *and* T. glabrescens *did not reduce cell migration to the joint, as well as the release of the chemoattractant chemokine CXCL1 or myeloperoxidase (MPO) activity in the periarticular tissue, at none of the assayed doses (data not shown). Since these extracts significantly inhibited TNF-*α* release* in vitro*, it is feasible to suppose that the absence of* in vivo* activity could be related to the low absorption or metabolization of the bioactive compounds.

**Figure 2:**
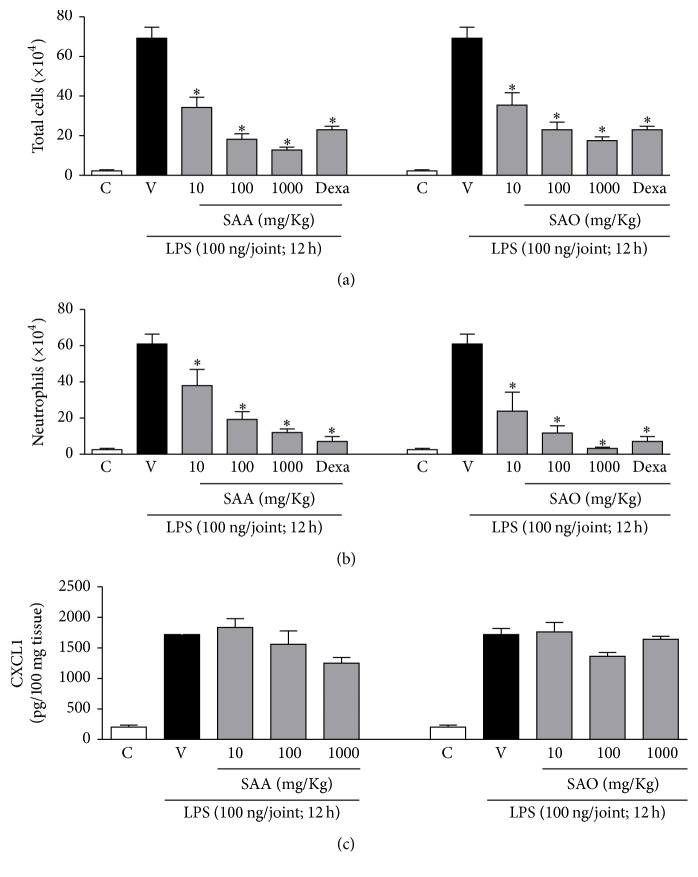
Treatments with the aqueous (SAA) and organic (SAO) fractions of* Stryphnodendron adstringens *reduce total cells and neutrophil recruitment into the knee joint during LPS-induced arthritis. The total number of cells (a) and numbers of neutrophils in the synovial cavity (b) were assessed 12 h after inflammation induction with LPS (vehicle-, V-, SAA-, and SAO-treated mice groups) or PBS (control: C) into the knee joints of mice. The concentration of CXCL1 (c) was assessed by ELISA 12 h after inflammation induction and expressed as pg/100 mg of tissue. Dexamethasone (Dexa, 4 mg/Kg) was used as control. All results are expressed as mean ± standard error of mean (SEM) and are representative of at least two experiments. ^*∗*^ *p* < 0.05 when compared to vehicle-treated mice (*n* = 6) (ANOVA, Student-Newman-Keuls posttest).

The organic (SAO) and the aqueous (SAA) fractions of* S. adstringens* significantly reduced leukocyte recruitment to the joint, at all assayed doses (Figure 2(a)). These fractions also reduced neutrophil accumulation into the joint cavity in a dose-dependent manner (Figure 2(b)). There was no reduction in CXCL1 concentration in the periarticular tissue (Figure 2(c)), as well as in TNF-*α* level and MPO activity after treatment with SAO or SAA (data not shown).

The cellular response to inflammation is initiated by neutrophils, which are the first effectors recruited in the acute inflammatory response. Monocytes are the next cells that migrate to the site of injury guided by chemotactic factors, following their differentiation into tissue macrophages [38]. Therefore, compounds capable of decreasing neutrophil migration may attenuate the inflammatory process and, for this reason, the* S. adstringens* fractions SAO and SAA are a source of potential anti-inflammatory compounds.

The anti-inflammatory activity of* S. adstringens* has been previously investigated. An acetone fraction of the stem bark reduced the paw edema induced by carrageenan and dextran in rats, given orally at 400 and 800 mg/kg. This fraction, administered to rats at 800 mg/kg, reduced the exudate volume and leukocyte migration in acute pleurisy induced by carrageenan and diminished the vascular permeability enhanced by the injection of acetic acid in the peritoneal cavity of mice, at the same dose. In addition, the fraction significantly reduced the paw edema in subplantar antigen-induced arthritis in rats, administered at 800 mg/kg [39]. We herein report the anti-inflammatory effect of* S. adstringens* at substantially lower doses and also demonstrate the activity of an aqueous fraction, constituted by highly polar constituents.

It is noteworthy that the administration of SAO and SAA at 100 mg/kg by oral route reduced leukocyte migration by 33 ± 11% and 26 ± 9%, respectively, a similar response as observed with the positive control dexamethasone given subcutaneously (33 ± 6% inhibition). This finding highlights the relevance of the anti-inflammatory effect of* S. adstringens* here reported. As far as we know, there is no previous report on the effect of* S. adstringens* on the acute arthritis model induced by LPS in mice knee, as well as* in vitro *data on the inhibition of TNF-*α* release.

Treatment with SAO and SAA markedly reduced the migration of neutrophils into the knee joint, but neither the content of CXCL1 nor the MPO activity was significantly diminished in the periarticular tissue. Hence, the decrease of neutrophil recruitment induced by these fractions probably does not result from CXCL1 inhibition and may be undergone by other mechanisms. CXC proteins, a class of CXCL1, are the major chemokines engaged in neutrophil recruitment to the inflamed tissue [40]. Neutrophil recruitment is also affected by the inhibition or production/release of other chemokines and by compounds interfering with chemokine receptors and the binding between chemokines and glycosaminoglycans, thus impairing neutrophil rolling. Decrease in expression or blockade of adhesion molecules found in neutrophils and endothelial cells may well affect the recruitment [41–44].

Myeloperoxidase (MPO) is an enzyme produced by neutrophils, which plays a major role in the generation of oxidative species, involved in host protection against microorganisms [45]. Despite the marked decrease in neutrophil counting in the joint of animals treated with SAO and SAA, we could not detect a significant reduction in MPO activity (data not shown).

*S. adstringens *extract reduced TNF-*α* production* in vitro* and it is feasible to suppose that this cytokine might also be in involved in the anti-inflammatory effect of SAO and SAA fractions. However, TNF-*α* contents could be neither quantified nor detected in the periarticular tissue of animals treated with the fractions, as well as in the control groups, 12 h after LPS administration. This time slot was set based on our previous results, which showed the peak of leukocyte migration into the inflamed joint to occur 12 h after LPS injection (unpublished data). Aiming at measuring TNF-*α* production* in vivo*, SAO and SAA were evaluated in another experiment and the periarticular tissue was collected six hours after injecting the inflammatory stimulus. Again, the cytokine could not be detected in any of the experimental groups (data not shown). Alternatively, we tried to measure TNF-*α* in the synovial fluid, since the cytokine has been reported to reach its maximal concentration in this medium two hours after LPS injection [46]. Therefore, the cytokine concentration was measured in washed-out synovial fluid samples collected one or three hours after LPS injection, employing a real-time PCR method. Nevertheless, there were no significant differences between the groups (data not shown).

It is not an easy task to quantify TNF-*α* in animals challenged with the intra-articular injection of LPS. Nakayama et al. [47] measured TNF-*α* contents by PCR in the synovia of interleukin 32-alpha (IL-32*α*) transgenic mice, two weeks after the intra-articular injection of LPS. The authors reported higher amounts of TNF-*α* in the transgenic mice in comparison to nontransgenic animals. The levels of TNF-*α* were quantified in the synovial fluid and in neutrophils of rats injected with LPS intra-articularly, two and three hours after stimuli, by flow cytometry [48]. The amount of synovial fluid and TNF-*α* contents are higher in rats than in mice. Such differences may explain the difficulties in detecting TNF-*α* in the present work, despite using distinct time slots and analytical methods.

The understanding of LPS signal transduction is crucial for the development of new anti-inflammatory agents. After LPS binding to toll-like receptor 4 (TLR4), a receptor complex consisting of dimerized TLR4 and MD-2 is formed and initiates a series of intracellular events, which depends on different sets of adapters. Both the early and late responses result in the activation of nuclear factor kappa B (NF-*κ*B) and led to the induction of cytokines, chemokines, and other transcription factors [49]. The inflammatory cells respond by increasing the production of inflammatory cytokines and chemokines involved in leukocyte recruitment. Therefore, compounds able to inhibit the production and/or release of these mediators may attenuate the inflammatory process and several plant species have been evaluated with this purpose, using LPS as inflammatory stimulus.

### 3.3. Phytochemical Characterization of SAO by Ultra Performance Liquid Chromatography (UPLC) Coupled to Electrospray Ionization Mass Spectrometry (ESI-MS)

**Figure 3:**
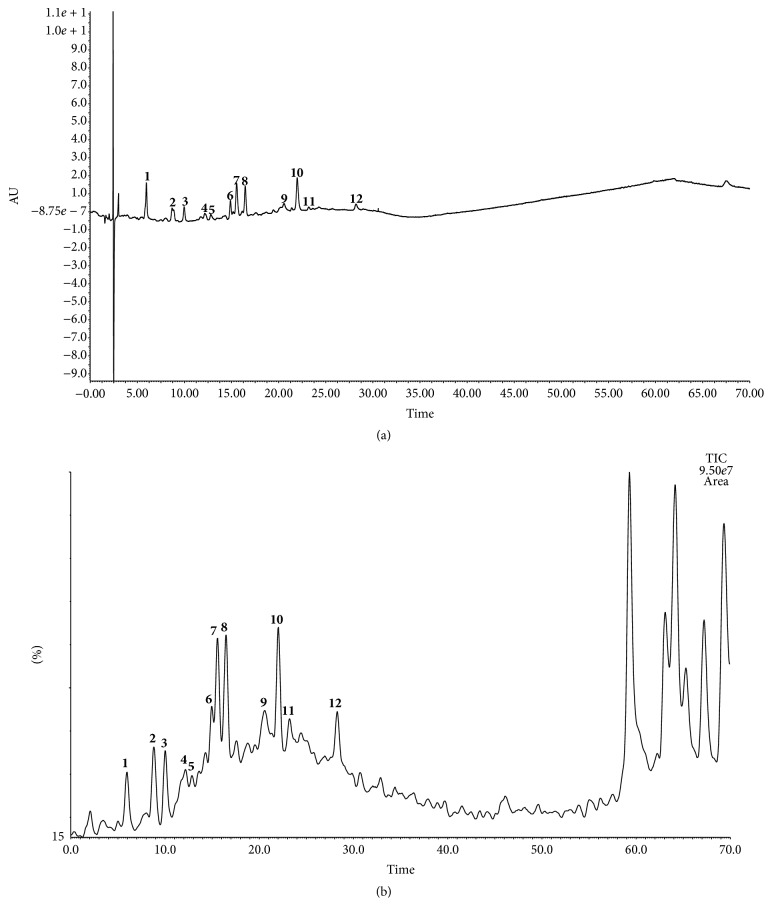
Chromatograms obtained for the organic fraction (SAO) of* Stryphnodendron adstringens*, registered by photodiode array detector (PDA) at 220 nm (a) and by electrospray ionization mass spectrometry (ESI-MS) in the negative ion mode of analysis (b). Identified peaks:** 1**, gallic acid,** 2**, dimer [two units of (epi)gallocatechin],** 3**, gallocatechin,** 4**, dimer [two units of (epi)gallocatechin gallate],** 5**, dimer [methyl-(epi)gallocatechin gallate and (epi)gallocatechin gallate],** 6**, epigallocatechin,** 7**, dimer [(epi)gallocatechin and (epi)gallocatechin gallate],** 8**, 4′-*O*-methyl-gallocatechin,** 9**, dimer [two units of (epi)gallocatechin gallate],** 10**, epigallocatechin 3-*O*-gallate,** 11**, dimer [methyl-(epi)gallocatechin and (epi)gallocatechin], and** 12**, methyl-(epi)gallocatechin gallate.

The chemical composition of* S. adstringens* organic fraction (SAO) was assessed by UPLC-ESI-MS. In the established conditions, 12 compounds were identified based on their UV spectral data and fragmentation pattern on ESI-MS and by comparison with reference compounds, when available, or with literature data reported for* S. adstringens *[50–52]. The chemical composition of the species comprises flavan-3-ol oligomers such as prodelphinidins and prorobinetinidins, along with derivatives. The assignments of the major peaks found in the UPLC-ESI-MS chromatogram of SAO (Figure 3) are depicted in Table 3.

The presence of gallic acid [**1**, retention time (Rt) = 5.96 min], gallocatechin (**3**, Rt = 9.98 min), epigallocatechin (**6**, Rt = 14.88 min), and epigallocatechin 3-*O*-gallate (**10**, Rt = 21.97 min) was inferred by comparison with the retention times and UV spectroscopic data obtained for authentic samples (data not shown). The other compounds described in Table 3 were ascribed based on their ESI fragmentation pattern, acquired in the positive and negative ion mode of analysis. The obtained data were compatible with monomers or dimmers of prodelphinidins, with or without substituents (methyl or galloyl units).

Prodelphinidins are condensed tannins of the B-type, formed by the linkage between successive flavan-3-ol units, usually between the C-4 position of the “upper” unit and the C-6 or C-8 position of the “lower” or “starter” unit [53]. The stereochemistry of the linkage may be either *α* or *β* originating different isomers and, for this reason, the unequivocal structure assignment based solely on MS data is not feasible.

**Figure 4:**
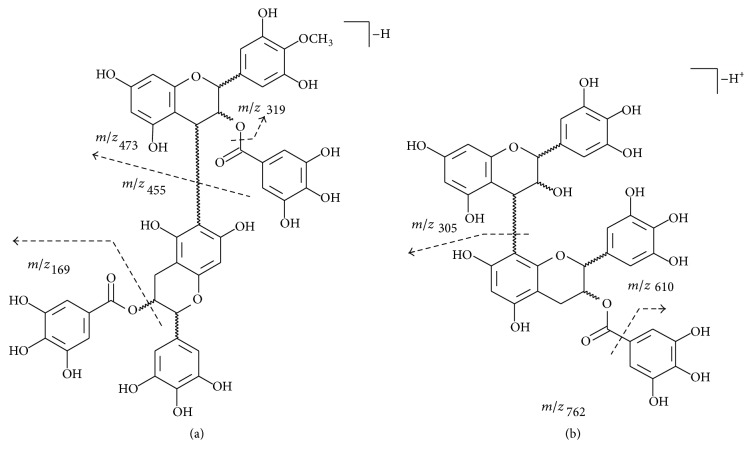
Proposal of fragmentation for compounds** 5** (a) [dimer of methyl-(epi)gallocatechin gallate and (epi)gallocatechin gallate] and** 7 **(b) [dimer of (epi)gallocatechin gallate and methyl-(epi)gallocatechin gallate].

In the negative ion mode of analysis (ESI^−^), the fragmentation of prodelphinidins is characterized by a peak at* m/z* 305, ascribed to a fragment resulting from the loss of either a gallocatechin or an epigallocatechin unit. Similarly, the presence of a peak at* m/z* 609 indicates a dimer composed of any of these units. In contrast, a fragment at* m/z* 169 indicates the loss of a galloyl unit from the compound. A scheme of fragmentation that generates the above-mentioned fragments is depicted in Figure 4 for compounds** 5** and** 7**.

**Figure 5:**
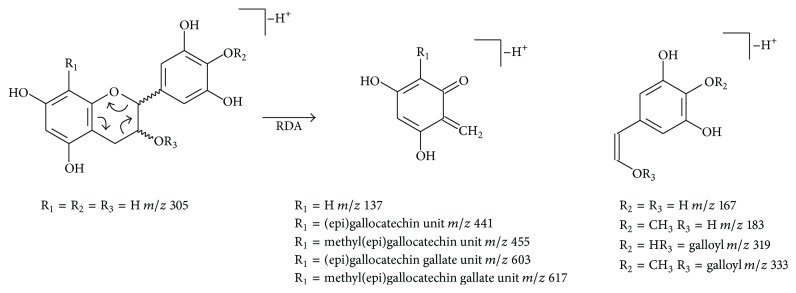
Characteristic fragments obtained from Retro-Diels-Alder (RDA) fission of a prodelphinidin.

Another characteristic fragmentation of prodelphinidins evolves from Retro-Diels-Alder (RDA) fission of the prodelphinidin nucleus, with the break of ring C to afford the fragment at* m/z* 137, a quinone methide [54] (Figure 5). The negative parent ion scan of* m/z* 137 was applied and allowed confirming that it results from the fragmentation of compounds** 4**,** 7**,** 8**,** 9**, and** 10**.

A previous investigation of an ethyl acetate fraction derived from the acetone/water (7 : 3 v/v) extract of* S. adstringens* stem bark led to the isolation of different prodelphinidins [50]. In the present study, we detected some peaks in the UPLC-ESI-MS chromatogram of SAO corresponding to compounds with molecular weight compatible with the prodelphinidins previously reported or their isomers, including 4′-*O*-methyl-gallocatechin (**8**), a dimer consisting of two units of (epi)gallocatechin (**2**, molecular weight - MW 610 g/mol), two dimers formed by two units of (epi)gallocatechin 3-*O*-gallate (**4** and** 9**, MW 914 g/mol), and a dimer composed of (epi)gallocatechin and (epi)gallocatechin 3-*O*-gallate (**7**, MW 762 g/mol).

Mello et al. [50] described the isolation of epigallocatechin 3-*O*-gallate-(4*β*→8)-epigallocatechin 3-*O*-gallate, a compound with a molecular weight of 914 g/mol. The analysis of SAO by UPLC-ESI-MS disclosed two peaks with distinct retention times in the chromatogram (**4**, Rt = 12.17 min, and** 9**, Rt = 20.57 min), but that gave the same* quasi*-molecular ion at* m/z* 913 [M − H]^−^. This finding suggests the occurrence of at least two isomers in the species. In addition, the ESI^−^-MS analysis of compound** 5 **(RT 12.84 min) gave a peak at* m/z* 927 [M − H]^−^, which was ascribed to a methylated derivative of one of the above-mentioned isomers. The location of the methyl group is suggested to be in ring B of the (epi)gallocatechin unit. This assumption was based on the fragmentation pattern of** 5**, which produced a peak at* m/z* 319, attributed to the residue of* O*-methyl-(epi)gallocatechin [M − H]^−^, as well as on the peak at* m/z* 169, attributed to the galloyl moiety [M − H]^−^. In case the galloyl moiety bears the methyl group, a fragment at* m/z* 183 should be found. Based on the available data, it is feasible to propose the structure of** 5** as a dimer of (epi)gallocatechin 3-*O*-gallate and* O*-methyl-(epi)gallocatechin 3-*O*-gallate (Figure 4(a)).

Analysis of the UPLC-ESI-MS spectrum of SAO also revealed a compound with MW of 624 g/mol (**11**, Rt = 23.25 min). The fragmentation pattern of** 11**, recorded in the negative ion mode of analysis, suggests that it is composed by one unit of (epi)gallocatechin (*m/z* 305) and one of 4′-*O*-methyl-(epi)gallocatechin (*m/z* 319). In contrast, compound** 12** gave the* quasi*-molecular ion at* m/z* 471 [M − H]^−^. This compound is probably a methylated (epi)gallocatechin gallate, supported by the presence of the fragments at* m/z* 169, corresponding to a galloyl group, and at* m/z* 319, ascribed to an unit of methyl-(epi)gallocatechin.

Prorobinetinidins were isolated from the acetone/water (7 : 3 v/v) extract of* S. adstringens* stem bark [51]. However, polyphenols of this class were not identified in the UPLC-ESI-MS analysis of SAO undertaken in the present work. Such absence could be related to distinct methods employed for preparing the crude extract and its derived fraction SAO, as well as to differences in the vegetal drugs employed in the studies.

Several prodelphinidins are biologically active and some have had their anti-inflammatory properties demonstrated by* in vitro *and* in vivo *models. Hence, epigallocatechin 3-*O*-gallate has been credited to possess anti-inflammatory properties that may affect the pathogenesis of different chronic inflammatory diseases. This compound is the major constituent of green tea and is probably one of the most studied proanthocyanidins. It inhibited TNF-*α* release by BALB/3T3 cells stimulated with okadaic acid (IC_50_ of 26 *µ*M) and suppressed the genes responsible for the expression of TNF-*α* [55]. Epigallocatechin 3-*O*-gallate, assayed at 50 *µ*g/mL, inhibited the release of TNF-*α* by LPS-stimulated THP-1 cells and the anti-*β*2GPI/*β*2GPI complex [56]. Epigallocatechin and epigallocatechin gallate decreased the production of interleukin 1 beta (IL-1*β*) and enhanced the production of IL-10 but had no effect on the production of IL-6 or TNF-*α*, when tested* in vitro* at 10 and 20 *μ*M in human leukocytes stimulated by LPS [57]. Dietary supplementation with 0.1% of epigallocatechin 3-*O*-gallate decreased the expression of genes that code the expression of proinflammatory cytokines in GK rats, including TNF-*α* and IL-1*β* [58].

The effect of prodelphinidins on the production of prostaglandin E2 (PGE2) by differentiated human chondrocytes was evaluated* in vitro*, as well as on the inhibition of cyclooxygenase isoenzymes COX-1 and COX-2. The synthesis of PGE2 was significantly reduced by dimers of gallocatechin and gallocatechin/epigallocatechin, along with a gallocatechin trimer, assayed at 10 and 100 *μ*g/L. Moreover, these compounds inhibited COX-1 and COX-2 [59].

Different dimers of prodelphinidins were identified by UPLC-ESI-MS analysis of the organic fraction of* S. adstringens* (SAO); therefore, based on the inhibition of inflammatory mediators previously reported for compounds of this class, it is feasible to suppose that prodelphinidins may contribute to the anti-inflammatory activity of SAO here described.

## 4. Conclusions

In conclusion, the* in vitro* screening of Brazilian plants on THP-1 cells stimulated by LPS allowed identifying four extracts with significant anti-TNF-*α* activity. The aqueous and the organic fractions derived from the ethanolic extract of* S. adstringens* stem bark showed marked anti-inflammatory activity on a model of acute arthritis induced by LPS, reducing cell migration to the periarticular tissue on a dose-dependent manner. Gallic acid and dimeric prodelphinidins of the B-type, identified by UPLC-ESI-MS analyses, are the compounds possibly responsible for the anti-inflammatory properties of the species. Our findings contribute to some extent to corroborating the traditional use of* S. adstringens* as anti-inflammatory and point out the potential of the species as a source of bioactive compounds.

## Figures and Tables

**Table 1:** Brazilian plants selected for study with their botanical and popular names, data of collection, voucher numbers, and ethnopharmacological uses.

Botanical name	Family	Location	Voucher number	Ethnopharmacological uses	References
*Bowdichia virgilioides *H.B.K.	Fabaceae	Belo Horizonte, MG	BHCB 166999	Diarrhea, gout, diabetes, bronchitis, hyperthermia, inflammation of the uterus, and rheumatism	[12, 13]
*Campomanesia lineatifolia *Ruiz & Pav.	Myrtaceae	*Campus* UFMG	BHCB 150.606	Dysentery, stomach and liver problems, diarrhea, problems of the urinary tract, and leucorrhea	[14, 15]
*Cordia guazumaefolia *(Desv.) Roem. & S.	Boraginaceae	*Campus *UFMG	BHCB 166998	Antirheumatic and anti-inflammatory agent	[16]
*Hymenaea courbaril *L.	Fabaceae	MHJB UFMG	BHCB 161553	Tonic, analgesic, antiseptic, anti-inflammatory, antipyretic, expectorant, antirheumatic, and laxative	[17]
*Hymenaea stigonocarpa *Mart. ex. Hayne	Fabaceae	*Campus* UFMG	BHCB 47.468		
*Licania tomentosa *Benth.	Chrysobalanaceae	*Campus* UFMG	BHCB 152221	Diabetes	[18]
*Mikania glomerata *Sprengel	Asteraceae	*Campus* UFMG	BHCB 167001	Asthma, bronchitis	[19]
*Paepalanthus bromelioides *Silveira	Eriocaulaceae	Serra do Cipó	BHCB 24241	Cytotoxic and antimutagenic	[20, 21]
*Stryphnodendron adstringens *(Mart.) Coville	Fabaceae	Ritápolis, MG	BHCB 111231	Wound healing and for the treatment of inflammation, diarrhea, and leucorrhea	[22]
*Stryphnodendron obovatum *Benth.	Fabaceae	*Campus* UCDB, MS	CGMS 329997		
*Terminalia glabrescens *Mart.	Combretaceae	*Campus* UFMG	BHCB 167015	Antimalarial, anticancer, and anti-inflammatory and for treatment of diarrhea, ulcers, and diabetes	[23]
*Vernonia phosphorea *(Vell.) Monteiro	Vernoniaceae	*Campus* UCDB, MS	CGMS 11970	Asthma, hemorrhoids, diabetes, and rheumatism and as diuretic and expectorant and for healing of wounds	[24]
*Vitex polygama *Cham.	Verbenaceae	*Campus* UFMG	BHCB 166996	Emmenagogue, diuretic, and treatment of renal diseases	[25, 26]

**Table 2:** Inhibition of TNF-*α* release by LPS-stimulated THP-1 cells induced by the ethanolic extracts of Brazilian plants.

Plant species	Plant part	Extraction yield (%)	Concentration (*µ*g/mL)	Inhibition of TNF-*α* (% ± SD)
Control			^*∗*^DMSO (0.1% v/v)	^*∗*^11.72 to 42.06 pg/mL
			^*∗*^LPS (100 ng/mL)	^*∗*^1523.96 to 3694.08 pg/mL
			Dexamethasone (0.1 *µ*M)	86.7 ± 1.1

*Bowdichia virgilioides*	Leaves	39.7	250	−6.9 ± 4.7
			125	−21.9 ± 13.6
			62.5	−46.0 ± 9.4
	Stem	34.2	250	−21.1 ± 6.4
			125	−118.2 ± 2.4
			62.5	−83.8 ± 9.7

*Campomanesia lineatifolia*	Leaves	37.8	250	41.1 ± 3.1
			125	4.9 ± 0.3
			62.5	−15.6 ± 0.6
	Stem	32.7	250	−78.2 ± 5.1
			125	−49.6 ± 8.8
			62.5	−37.2 ± 7.1

*Cordia guazumaefolia*	Leaves	22.1	250	ND
			125	ND
			62.5	ND
	Stem	18.8	250	ND
			125	ND
			62.5	ND

*Hymenaea courbaril*	Leaves	13.8	250	98.6 ± 1.5
			125	28.6 ± 9.7
			62.5	98.6 ± 23.7

*Hymenaea stigonocarpa*	Leaves	8.8	250	98.5 ± 0.6
			125	35.5 ± 6.0
			62.5	−11.9 ± 0.9

*Licania tomentosa*	Leaves	29.4	250	−21.9 ± 4.6
			125	−60.8 ± 10.1
			62.5	−70.2 ± 7.1

*Mikania glomerata*	Leaves	16.0	250	12.3 ± 12.3
			125	−17.8 ± 2.7
			62.5	−20.4 ± 2.3
	Stem	18.7	250	ND
			125	ND
			62.5	ND

*Paepalanthus bromelioides*	Leaves	7.7	250	−36.4 ± 15.9
			125	−18.5 ± 53.2
			62.5	−25.6 ± 24.7

*Stryphnodendron adstringens*	Bark	44.8	250	42.1 ± 3.1
			125	19.3 ± 0.5
			62.5	−9.4 ± 0.8

*Stryphnodendron obovatum*	Bark	33.1	250	54.2 ± 3.0
			125	31.6 ± 1.8
			62.5	4.4 ± 0.1

*Terminalia glabrescens*	Leaves	26.3	250	42.2 ± 5.3
			125	26.5 ± 3.0
			62.5	−0.9 ± 0.1
	Stem	29.1	250	−5.3 ± 11.3
			125	−8.31 ± 41.5
			62.5	−16.8 ± 28.2

*Vernonia phosphorea*	Leaves	31.2	250	96.2 ± 1.1
			125	13.8 ± 0.7
			62.5	−23.6 ± 0.4

*Vitex polygama*	Leaves	36.4	250	−35.3 ± 6.3
			125	−93.2 ± 3.1
			62.5	−66.7 ± 9.0
	Stem	32.1	250	−56.7 ± 6.6
			125	−48.2 ± 6.9
			62.5	−36.4 ± 2.3

^*∗*^Concentrations of TNF-*α* measured in the control (0.1% DMSO) or LPS-stimulated cells.

ND: not determined (cell viability < 90%).

**Table 3:** Peak assignments and UPLC-ESI-MS fragmentation data obtained for the constituents of the organic fraction of *Stryphnodendron adstringens *(SAO).

Number	Postulated compounds	Rt (min)	*λ* max (nm)	ESI^−^ ions (*m*/*z*)	ESI^+^ ions (*m*/*z*)	MW (g/mol)	Molecular formula
1	Gallic acid	5.96	212.3; 269.3	169.4 [M − H]^−^, 125.1	—	170	C_7_H_6_O_5_
2	Dimer: two units of (epi)gallocatechin	8.70	271.0	609.5 [M − H]^−^, 137.2, 209.1, 305.3, 423.3, 441.6	611.7 [M + H]^+^, 142.1, 155.2, 187.2	610	C_30_H_26_O_14_
3	Gallocatechin	9.98	271.0	305.4 [M − H]^−^, 137.0, 165.2	307.5 [M + H]^+^, 142.1, 164.2, 187.2	306	C_15_H_14_O_7_
4	Dimer: two units of (epi)gallocatechin 3-*O*-gallate	12.17	271.0	913.6 [M − H]^−^, 137.1, 169.2, 259.2, 382.1, 445.3, 761.5	915.6 [M + H]^+^, 164.2	914	C_44_H_34_O_22_
5	Dimer: 4′-*O*-methyl-(epi)gallocatechin 3-*O*-gallate and (epi)gallocatechin 3-*O*-gallate	12.84	271.0	927.5 [M − H]^−^, 137.2, 169.3, 305.1, 319.5, 609.2, 762.5	164.2, 930.4	928	C_45_H_38_O_22_
6	Epigallocatechin	14.88	271.0	305.4 [M − H]^−^, 137.2, 167.1, 179.2	307.6 [M + H]^+^, 139.2, 164.4	306	C_15_H_14_O_7_
7	Dimer: (epi)gallocatechin and (epi)gallocatechin 3-*O*-gallate	15.54	271.0	761.5 [M − H]^−^, 137.3, 169.0, 305.1, 591.4, 609.9	763.5 [M + H]^+^, 164.1, 208.4	762	C_37_H_30_O_18_
8	4′-*O*-Methyl-gallocatechin	16.50	271.0	319.3 [M − H]^−^, 137.0, 181.2, 220.2, 261.2, 305.3	321.3 [M + H]^+^, 164.4	320	C_16_H_16_O_7_
9	Dimer: two units of (epi)gallocatechin 3-*O*-gallate	20.57	271.0	913.4 [M − H]^−^, 137.0, 169.2, 319.6, 458.2, 608.7, 775.5	915.8 [M + H]^+^, 164.3, 187.3, 317.1	914	C_44_H_34_O_22_
10	Epigallocatechin 3-*O*-gallate	21.97	271.0	457.3 [M − H]^−^, 137.2, 169.3, 305.3, 319.1, 331.4	459.5 [M + H]^+^, 164.4, 289.4	458	C_22_H_18_O_11_
11	Dimer: 4′-*O*-methyl-(epi)gallocatechin and (epi)gallocatechin	23.25	271.0	623.3 [M − H]^−^, 137.0, 305.2, 319.6, 440.8	624.6 [M + H]^+^, 164.4	624	C_31_H_30_O_14_
12	4′-*O*-Methyl-(epi)gallocatechin 3-*O*-gallate	28.20	271.0	471.7 [M − H]^−^, 137.1, 169.1, 319.3	473.4 [M + H]^+^, 164.3, 187.3, 208.5	472	C_23_H_20_O_11_
